# Physical activity and fat mass gain in Mexican school-age children: a cohort study

**DOI:** 10.1186/1471-2431-12-109

**Published:** 2012-07-28

**Authors:** Alejandra Jáuregui, Salvador Villalpando, Eduardo Rangel-Baltazar, Yaveth A Lara-Zamudio, Marcia M Castillo-García

**Affiliations:** 1 Division of Nutrition and Health, National Institute of Public Health of Mexico, Av Universidad 655 Col Sta Ma Ahuacatitlán, Cuernavaca, Morelos, C.P. 62100, Mexico

## Abstract

**Background:**

In México, the prevalence of unhealthy weight increased from 24% at 6 y to 33% at 12 y of age, opening a window of opportunity to better understand the pathogenesis of obesity. The objective of this study was to explore the association between time spent on medium, vigorous physical activity (MVPA) and concurrent gains in BMI, fat mass (FM) and fat-free mass (FFM), alternately, in a cohort of Mexican children followed from kindergarten (baseline) to 2nd grade elementary school (endline).

**Methods:**

The MVPA (5-d accelerometry), BMI, FM and FFM (air displacement plethysmography) were measured at baseline and endline. Associations between gains in BMI, FM and FFM and changes in MVPA were examined using lagged and dynamic regression models, controlling for energy intake and demographic variables.

**Results:**

A total of 205 children were analyzed. Gender affected the effect of MVPA on FM gain. In girls, a high baseline MVPA predicted a lower FM gain (-0.96 kg, p=0.025) compared to low/medium MVPA. Increasing, decreasing or having a persistently high MVPA predicted a lower FM gain (range -1.6 to -1.03 kg, p<0.05) compared to persistently low MVPA. In boys, increases in MVPA were associated with higher gains in BMI (+0.76 kg/m2, p=0.04) and FFM (+1.1 kg, p=0.01) compared to persistently low MVPA.

**Conclusion:**

These results support a protective role of MVPA on FM gain in girls, suggesting that it may play a crucial role in the development of obesity. Further research on the gender effect of MVPA is warranted to better understand its role in the prevention and control of overweight and obesity.

## Background

Childhood obesity is an alarming public health problem worldwide [1]. The negative consequences of obesity include short and long-term social and economic consequences and emotional and pathophysiologic complications at individual level [2,3]. In México, the summed prevalence of overweight and obesity in children was 24.3% at 6 y of age and 32.5% at 12-y of age [4], representing a difference of 12.2 percentage points, the largest during elementary school.

Lack of physical activity is assumed to be an important contributing factor in the development of childhood obesity [5,6]. Many studies have investigated the association between objectively measured physical activity and obesity in children; however results have been inconsistent [7,8], probably due to inadequate measurements of physical activity and body composition [9] or study design flaws [8]. It has been proposed that physical activity intensity may be influential on body composition [10,11]. The WHO has recommended that children should accumulate at least 60 min/d of moderate-vigorous physical activity (MVPA) [12]. Studies evaluating the relationship between MVPA and fat mass (FM) in children have suggested a negative association [13-16]. Whether an association between MVPA and FM exists during childhood is yet to be confirmed.

Despite global efforts to curb the obesity epidemic in children, there are very few examples of successful group interventions aimed to prevent and control it [17,18]. The school age years could be a crucial time to study the determinants of childhood obesity since it is the period of the “adiposity rebound” [19], when body mass index (BMI) increases after reaching its nadir in childhood. This prospective longitudinal study aims to explore the association between time spent on MVPA and concurrent gains in BMI, fat mass (FM) and fat-free mass (FFM) in a cohort of children in transit from kindergarten to elementary school.

## Methods

### Subjects and study design

The cohort was assembled with healthy children 5 to 6 years old at the beginning of the study, recruited from a convenience sample of five kindergartens located in high, middle and low socioeconomic status neighbourhoods in Cuernavaca, Mexico. The number of children attending each kindergarten varied from 120 to 310. Inclusion criteria were being healthy, according to report of mothers or care-takers, being free of chronic diseases or physical impediments affecting physical growth, and having adequate feeding or anthropometric measurements.

In México, kindergartens have three grades. Once students graduate, they may attend different elementary schools. Parents registering children for the last grade of kindergarten were invited to participate in the study. After discussing the objectives and risks of the study, parents signed an informed consent; before initiating data collection, children were asked for their assent. Measurements were carried out at the last grade of kindergarten (2004, baseline) and the second elementary school grade (2006, endline). After graduating from kindergarten, children were dispersed into 36 elementary schools.

The protocol was reviewed and approved by the Ethics, Biosecurity and Research Committees of the National Institute of Public Health, Cuernavaca, Mexico. Education authorities gave their authorization for the study.

### Demographics

Demographic information was collected using a questionnaire administered to the mother or caretaker [20], validated in Mexican population [21]. An indicator of socioeconomic status was constructed using a principal component analysis [22] based on household characteristics (flooring material, ceiling, walls, water source, sewage and number of domestic appliances). The first component explained 40.4% of the total variance, with a Kaiser-Mayer-Olkin measure of sampling adequacy = 0.83. This component, divided into tertiles, was used as a proxy for low, medium and high socioeconomic status categories [20].

### Physical Activity

Physical activity was measured using RT3 accelerometers (StayHealthy Inc., Monrovia, CA, USA). This motion sensor detects body movement in three planes (X, Y and Z) and integrates acceleration into a single value called “monitor vector” or “counts”. The RT3 accelerometer has previously been validated to provide information on physical activity intensity in adults [23] and children [23,24]. Children wore the accelerometers during five full days (3 weekdays plus 2 weekend days), attached to the right hip by a small denim sack and a belt. They were instructed to wear the accelerometer during waking hours, and take it off only for showering or water sports. The accelerometer was programmed to record activity counts in 1-min epochs. It has been suggested that if activity intensity need only be classified as time spent in moderate-intensity activity or more, a 1 min epoch setting may give a full picture of activity [25]. Several studies have used this epoch when measuring physical activity in preschool and school age children [26-28].

The processing of physical activity counts has been reported elsewhere [29]. Briefly, individual accelerometry records of at least 10 h/d, from 07:00 to 23:00 hrs, were considered as valid for the analysis. Children wore the accelerometers for a mean of 4 d and 13.6 h/d. Cases with at least one valid day of physical activity measurements for each measurement wave were included. Minutes of MVPA were calculated using the cut-off point proposed by Rowlands for children >9 years old (970.2 counts/min) [23], as it is comparable to the cut-off proposed for Chilean preschool children [30].

Since physical activity has been demonstrated to be dependent on sex and age in this cohort [29] and other studies [31,32], subjects were divided into tertiles according to the minutes spent in MVPA by sex and school grade. Tertiles 1 and 2 of MVPA were merged into one category (medium/low), because initial analyses showed no differences between the two. Changes in MVPA, from baseline to endline, were grouped in four categories: 1) persistently high MVPA, 2) persistently medium/low MVPA, 3) decreasing from the high to the medium/low MVPA category, and 4) increasing from the medium/low to the high MVPA category.

### Anthropometry

Weight and height were measured using standard techniques [33]. The height of children was measured to the nearest mm using a stadimeter (Shorr Productions, Olney, Maryland, USA), and body weight using an electronic scale (Model BWB-627-A, Tanita Corporation, Japan). BMI was calculated (kg/m2).

### Fat mass and fat free mass

Body composition was measured using air displacement plethysmography (BOD POD; Life Measurement Inc., Concord, CA, USA). BOD-POD was calibrated before each measurement, using a 49.273 L cylinder. Children were tested wearing minimal tight-fitting clothing (swimming suit) and swimming cap to compress the hair [34,35]. Volume of thoracic capacity was used to correct body volume (corrected body volume = total body volume - thoracic capacity). Body density was calculated as body mass divided by corrected body volume [36]. FM (in kg) was calculated using the equation of Siri [36]. Fat-free mass (FFM, in kg) was calculated subtracting FM from body weight.

### Dietary intake data

During five days, a 24 h dietary recalls was collected on children, from the mother or caretaker by trained personnel. Energy and nutrient intake were derived from food composition tables compiled by the National Institute of Public Health [37]. Energy intakes greater than five standard deviations from their respective means were excluded from the analysis as not plausible.

### Data analysis

Descriptions were made using means and SD, differences among descriptive variables were assessed using Student *t*-tests for independent variables.

Multiple linear regression methods were constructed to test the effects of changes in MVPA category on changes in BMI, FM or FFM, alternately. Two different approaches were used: lagged and dynamic regression models [38]. A lagged linear regression model was used to assess the effect of baseline MVPA category on gains in BMI, FM or FFM, between baseline and endline represented by the following equation:

(1) $$\Delta {\text{BMI}}_{\text{e}-\text{b}}=\beta_{0}+\beta_{1}{\text{BMI}}_{\text{b}}+\beta_{2}{\text{PA}}_{\text{b}}+\beta_{3}{\text{covariates}}_{\text{b}}+\beta_{4}\Delta {\text{covariates}}_{\text{e}-\text{b}}+E$$

This model estimated the effect of baseline MVPA category on gains in BMI, FM or FFM controlling for baseline BMI, FM or FFM, energy intake and height, age, gender, socioeconomic status, and changes in daily energy intake and height for the same period and for the clustered design of the study. An interaction term was included to evaluate whether the association between baseline MVPA category and gains in BMI, FM or FFM, alternately, differed by sex.

A separate lagged regression model of minutes engaged in MVPA at baseline and FM gain, adjusting by the above confounding variables and interaction, was conducted to estimate the magnitude of the associations.

A dynamic model was used to assess the effect of the changes in MVPA category on the gains in BMI, FM or FFM, alternately. These latter variables were modelled as a function of MVPA changes (4-category variable), adjusted by the same covariables and interaction used in the lagged model:

(2) $$\Delta {\text{BMI}}_{\text{e}-\text{b}}=\beta_{0}+\beta_{1}{\text{BMI}}_{\text{b}}+\beta_{2}\Delta {\text{PA}}_{\text{e}-\text{b}}+\beta_{3}\text{time}\;\text{invariant}\;{\text{covariates}}_{\text{b}}+\beta_{4}\Delta \text{time}\;\text{varying}\;{\text{covariates}}_{\text{b}}+\beta_{5}\Delta \text{time}\;\text{varying}\;{\text{covariates}}_{\text{e}-\text{b}}+E$$

Differences between unadjusted means were considered significant if p < 0.05; for associations in regression models, if p < 0.05 for main effects and p < 0.1 for interactions [39]. Data were analyzed using STATA software, version 11.0 (StataCorp, College Station, Texas, USA).

## Results

**Figure 1:**
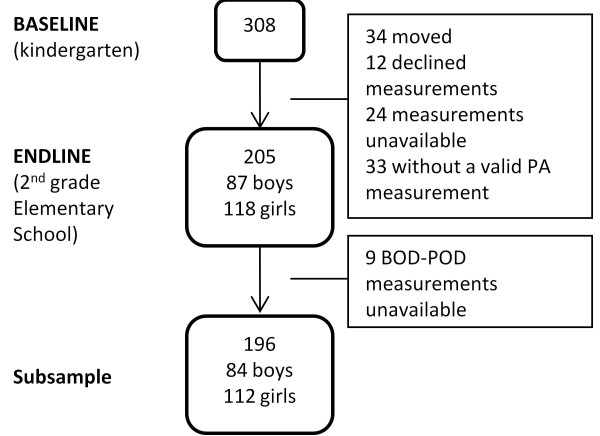
**Consort of the follow-up of the cohort.** A total of 205 children from Cuernavaca, Mexico were followed up between March 2004 and October 2006.

The analytical sample included 205 children (118 girls and 87 boys) out of the 308 originally recruited; 15.6% were lost to follow-up (n = 46) because of moving out the neighbourhood (n = 34) or refusing to participate (n = 12). Additionally, 57 cases were excluded from the analysis, due to incomplete data. A subsample (n = 196) with available BOD-POD measurements was assembled out of the 205 children in the analytical sample (Figure 1).

No differences were found among most demographic and anthropometric characteristics, however, the proportion of boys resulted significantly lower in the analytical sample (42%) than in the lost-to-follow-up or excluded children (61%, p < 0.0001). No differences were found between the BOD-POD subsample and the original cohort. Within the analytical sample, no differences were found between boys and girls with respect to baseline age, weight, height and BMI; however girls had a higher FM and boys a higher FFM, energy intake and MVPA (p < 0.05) (Table 1)..

**Table 1:** Characteristics of the cohort, stratified by gender

**Variable**	**Boys**		**Girls**	
	**n = 87**		**n = 118**	
	**Baseline**	**Endline**	**Baseline**	**Endline**
Age (y)	5.9 (0.4)	8.1 (0.3)	6.0 (0.4)	8.1 (0.3)
Height (cm)	114.1 (5.3)	126.6 (6.0)	113.4 (6.1)	125.7 (6.8)
Weight (kg)	21.4 (4.3)	28.5 (7.1)	20.8 (4.7)	28.2 (7.2)
BMI (kg/m^2^)	16.3 (2.5)	17.6 (3.1)	16.1 (2.5)	17.6 (3.2)
Fat mass (kg)^1^	4.6 (2.8)	7.9 (4.3)	5.5 (2.9) ^*^	8.2 (4.5)
Fat free mass (kg)^1^	16.8 (2.0)	20.6 (3.4)	15.3 (2.3) ^*^	19.8 (3.2)
Energy intake (kcal/d)	1547 (350)	1632 (338)	1443 (345) ^*^	1539 (338)
MVPA (min/d)	163 (58)	143 (53)	125 (45) ^*^	98 (46)

Values are means (SD).

Abbreviations: MVPA = moderate-vigorous physical activity.

^1^Means calculated for BOD-POD subsample (n = 84 boys, 112 girls).

* P value < 0.05 for Student’s *t* test comparing boys and girls

Between baseline and endline, 15.6% of children (N = 32) remained with a high MVPA, 49.8% (N = 102) with a medium/low MVPA, 17.1% (N = 36) changed to a higher MVPA category and 17.6% (N = 35) changed to a lower MVPA category (Data not shown). Increases in the mean body weight (+7.3 kg), height (+12.4 cm) and BMI (+1.4 kg/m2) occurred from baseline to endline. Compared with the mean for the WHO-2007 growth reference [40], children in the analytical sample had higher baseline BMI (16.2 kg/m2, 75th centile). This deviation was more pronounced at endline (17.6 kg/m2, 85th centile).

### Lagged models

The lagged model of FM gain (Table 2) showed a different effect of baseline MVPA category on gains in FM between boys and girls (p < 0.05). Girls with a high baseline MVPA had a lower FM gain by −0.92 kg compared to girls with a low/medium baseline MVPA (p < 0.05), but no differences in FM gain were found in boys. The lagged model of minutes engaged in MVPA at baseline and FM gain (Table 2) predicted that an increase of ten minutes in baseline MVPA was associated with a lower FM gain by −0.12 kg (p < 0.05) in girls; the effect in boys was not significant. No associations were found between baseline MVPA category and gains in BMI or FFM adjusting for confounding variables (Data not shown)..

**Table 2:** **Lagged models**^**1**^**of FM gain by baseline MVPA category or time engaged in MVPA**

	**ΔFM (kg) N = 196 (Boys = 84, Girls = 112)**	
	**β**	**95%CI**
**Model 1. Baseline MVPA category** (medium/low MVPA as reference)		
Girls ^2^	−0.92	−1.34, -0.49*
Boys	−0.05	−0.86, 0.75
**Model 2. Time engaged in baseline MVPA (10 min/d)**		
Girls ^3^	−0.12	−0.21, -0.04*
Boys	0.03	−0.01, 0.01

Abbreviations: MVPA = moderate-vigorous physical activity, ΔFM = fat mass gain, β = regression coefficient, CI = confidence interval.

^1^ Adjusted by initial fat mass, energy intake and height, age, gender, socioeconomic status and changes in energy intake and height. Grouping variable: School affiliation during the first survey. R2 for model = 0.19.

^2^ P value of baseline MVPA category x gender interaction: 0.046.

^3^ P value of the time engaged in baseline MVPA x gender interaction: 0.09.

*P value < 0.05

### Dynamic models

**Figure 2:**
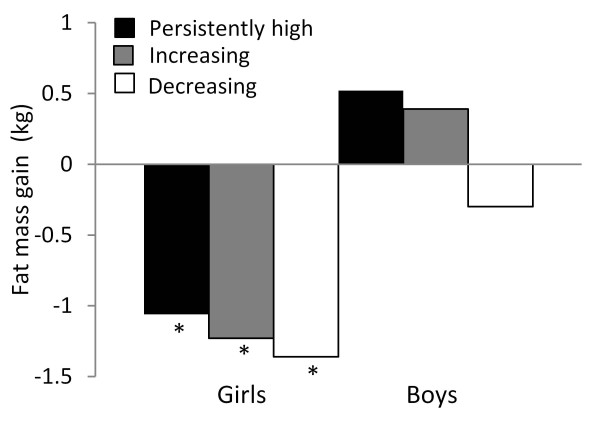
**Adjusted fat mass gain (kg) by changes in MVPA category.** The comparison term was persistently low MVPA . Adjusted in a multiple linear regression model for initial FM, energy intake, and height, age, gender, socioeconomic status and changes in energy intake and height. School affiliation at baseline is the grouping variable. N = 196 (Boys = 84, Girls = 112), R2 = 0.20. *p < 0.05.

The dynamic models showed no differences in the effect of categories of MVPA change on gains in BMI or FFM between boys and girls. However boys increasing their MVPA had a BMI gain of 0.76 kg/m2 (p = 0.04) and a FFM gain of 1.1 kg (p = 0.01) higher than their peers with a persistently low MVPA (Table 3); such a difference was not found in girls. The dynamic model of FM gain showed that the effect of increasing or having a persistently high MVPA was different between boys and girls (p < 0.05) (Table 3). Girls decreasing, increasing or having a persistently high MVPA, presented a FM gain of approximately −1 kg compared to those with a persistently low MVPA (p < 0.05). In boys, the adjusted effect on FM gain was not different among categories of MVPA change (Figure 2).

**Table 3:** **Dynamic models**^**1**^**of gains in BMI, FFM or FM by category of physical activity change. Persistently medium/low MVPA is the reference**

	**Decreasing**		**Increasing**		**Persistently high**	
	**β**	**95%CI**	**β**	**95%CI**	**β**	**95%CI**
**ΔBMI model** N = 205 (Boys = 87, Girls = 118)						
Girls	−0.19	−0.77, 0.39	−0.22	−2.02, 1.59	−0.25	−0.91, 0.40
Boys	0.16	−0.89, 1.21	0.76	0.06, 1.44*	0.41	−0.15, 0.97
**ΔFFM model** N = 196 (Boys = 84, Girls = 112)						
Girls	0.16	−0.45, 0.77	0.08	−1.36, 1.52	0.43	−0.11, 0.97
Boys	0.67	−0.39, 1.72	1.10	0.37, 1.84*	0.20	−1.43, 1.84
**ΔFM model** N = 196 (Boys = 84, Girls = 112)						
Girls ^2^	−1.36	−1.93, -0.79**	−1.23	−2.14, -0.33*	−1.06	−1.62, -0.50**
Boys	−0.30	−1.77, -1.18	0.39	−0.75, 1.53	0.52	−0.59, 1.62

Abbreviations: MVPA = moderate-vigorous physical activity, ΔFM = fat mass gain, ΔFFM = fat free mass gain, ΔBMI = BMI gain β = regression coefficient, CI = confidence interval.

^1^ Adjusted by initial BMI, FM or FFM, respectively, and initial energy intake and height, age, gender, socioeconomic status and changes in energy intake and height. Grouping variable = School affiliation during the first survey. R2 for models: BMI = 0.18; FFM = 0.20; FM = 0.55.

^2^ P value of increasing x gender interaction: 0.044; P value of persistently high x gender interaction: 0.048.

Level of statistical significance: * P value < 0.05; ** P value < 0.01.

## Discussion

Results of this research present evidence that gender affected the effect of MVPA on FM gain, but not BMI or FFM gain. A high baseline MVPA predicted a lower FM gain in girls. Findings also support that even though gender did not influence the effect of MVPA on gains in BMI or FFM, boys increasing MVPA demonstrated higher gains in these variables.

The size of the effect of MVPA on FM gain was biologically relevant: for each additional 10 min spent in MVPA at baseline FM gain was reduced by −0.12 kg at endline. So that theoretically, if the recommendation of 60 min/d of MVPA was met [12], gains in FM could be reduced by −0.72 kg. This number is very close to the figures obtained in this analysis for the most active girls, who gained −0.92 kg less FM at endline. Other studies reported a similar association. In a cohort of American children 10 minutes of MVPA at age 5 y resulted in 0.2 kg less FM at age 8 and 11 y [41]. In a different American cohort, children with low preschool physical activity levels gained substantially more FM during follow-up than their more active counterparts [42].

In the present study, the lagged models do not evaluate any baseline/endline change in MVPA, therefore, the observed reduction in FM gain could be attributed to either initial MVPA or changes in MVPA level during follow-up. The same type of association was confirmed in a dynamic regression model but it was significant only for girls. The effects of persistently high (β = −1.06 kg), increasing (β = −1.23 kg) or decreasing (β = −1.36 kg) MVPA category (dynamic model) were similar to the effect seen for baseline high MVPA (β = −0.92 kg, lagged model) on FM gain. These results suggest that having a high MVPA at any time has a protective role against FM gain. Several studies have evaluated the associations between changes in physical activity with changes in adiposity. In an 8-month follow-up of Chinese school-aged children, girls with the highest MVPA had less increases in body fatness (−0.5%) [13]. Another longitudinal study in French children found no associations between baseline physical activity and changes in adiposity; however girls decreasing their physical activity level demonstrated higher adiposity gains. [43] An 8 y follow-up study in children reported that higher accumulated physical activity was associated with less body fat at later age [44]; however predictions of initial physical activity level on adiposity changes were not investigated.

The gender difference in the effect of MVPA on FM gain was also found in previous studies, but results were more consistent during pubertal age [43,45,46] than in childhood [41,44]. The endline age of children in this study varied from 8–9 y, at which an undetermined proportion of girls may have already started their pubertal spurt, making them more susceptible to FM accumulation and therefore to a stronger protective role of MVPA. Though, sexual development was not evaluated. The difference in the effect of MVPA could be also explained by a reported threshold of 115–120 min/d of MVPA to detect impact of physical activity on FM [10,47]. At endline, boys in the low/medium MVPA category of the study barely reached this threshold (MVPA = 113.0 min/d, 95%CI = 104.4, 121.5), and girls were far below it (MVPA = 75 min/d, 95%CI = 66.0, 77.5, data not shown). Finally, other studies have explained similar gender differences by a stronger effect of dietary energy intake on FM, than the influence of physical activity; [48] in this analysis, regressions were controlled for dietary intake which did not show any positive association.

In our sample, boys increasing MVPA category gained more BMI and FFM compared to their peers with a persistently low MVPA. These findings are consistent with the results of a physical activity intervention in 6–8 y boys which found an increase of total body and regional lean mass in exercised boys [49]. This unexpected association suggests that the effect of MVPA on body components depends upon gender differences in baseline body composition. In our sample, boys had a significantly larger FFM and a smaller FM than girls. Muscle remodelling occurs rapidly in response to physical activity and protein intake [50], thus, it can be speculated that the larger increases in BMI and FFM seen in boys in higher MVPA categories are due to enhancement of muscle mass following remodelling induced by a more intense physical activity.

The main strengths of this study include its longitudinal design, providing robustness to causality implications. The study is based on robust and objective measurement of adiposity and physical activity and the statistical models were adjusted for known potentially confounding variables. The MVPA categories were stratified by age and gender to account for gender differences and age-related change in activity over time [31,32]. Finally we demonstrated that losses-to-follow-up did not bias the results. However, some limitations are recognized. Residual confounding may remain. Accelerometers are unable to measure water sports such as swimming and underestimate weight bearing activities such as cycling or climbing stairs. There is a possibility of misclassification in the four categories of change in MVPA. A single 5-d accelerometry measurement might not accurately represent variations in MVPA along follow-up. Therefore a non-systematic misclassification could have occurred among the four categories. In that case, the expected differences between categories could be larger. Another limitation is the fact that sexual development was not evaluated. Since girls are more susceptible to FM accumulation [51], results in girls of the effect of MVPA in FM gain herein presented could be lower.

## Conclusions

Results from this work support a protective role of MVPA on FM gain, suggesting that it may play a crucial role in the development of obesity in girls. Given that physical activity tracks throughout childhood [52], it is important to establish an active lifestyle early in life to prevent fat accumulation, particularly in girls. Further research evaluating the gender difference in the effect of MVPA on FM gain is warranted to better understand its role in the prevention and control of overweight and obesity. Findings from this study may have programmatic implications to improve physical activity interventions aiming to reduce and prevent obesity.

## Abbreviations

MVPA, Moderate - Vigorous Physical Activity; BMI, Body Mass Index; FM, Fat Mass; FFM, Fat free mass.

## Competing interests

The authors declare that they have no competing interests.

## Authors’ contributions

AJM performed the statistical analysis, data interpretation and drafted the manuscript. SV conceived of the study, participated in its design and coordination and helped to draft the manuscript. ERB, MMCG and YALZ participated in the design and acquisition of data and revised the manuscript critically for intellectual content. All authors read and approved the final manuscript.

## Pre-publication history

The pre-publication history for this paper can be accessed here:

http://www.biomedcentral.com/1471-2431/12/109/prepub
